# To Dr. Batty

**Published:** 1804-01-01

**Authors:** M. Baillie

**Affiliations:** Grosvenor Street


### To Dr. BATTY.

Dear Sir,

THE enclosed Case has been sent to me by a very sensible practitioner in the country, Mr. Blagden, surgeon, of Petworth, who wishes that it may be communicated to the public. It occurs, I believe, very rarely, is described with singular perspicuity, and I trust that you will think it not unworthy of a place in the Medical Journal.

*Grosvenor Street,* I am, &c.

*Dec.* 5, 1803. M. BAILLIE.

---

On the 28th of August last, I attended the wife of a reputable person in this neighbourhood, in her second labor. The first, which happened four years before, was, from the smallness of the inferior aperture of the pelvis, an extremely laborious one; the present was less so, though the last five or six pains were unusually violent: however, the labor was in no other respect uncommon, except that the patient spoke of a greater degree of suffering not only during but also between those pains in the right, than in the left side of the uterus, although, in the last months of utero-gestation, the abdomen had been more full on the left than on the right side. The placenta was expelled with ease.

After waiting the usual time I returned home, about one in the morning; but was called again, in haste, in less than half an hour. The patient was laboring under violent and unceasing bearing-down pain, and strongly impressed with the idea that she had another child. On examination, I found a monstrous swelling of the right labium, extending itself to the perinæum. I endeavoured, by fomentations and the repeated use of opiates, to give the poor woman some ease, but without the smallest success; the size of the tumour increased till it equalled a child's head, and the pain likewise increased till it exceeded every degree of it which I had before witnessed, and might be truly called torture.

In less than three hours from the commencement of this affection, the tumour began to grow discoloured, and within another hour the most prominent part of it assumed a livid hue. After reflecting with all the coolness of which so unusual and alarming a situation admitted, it occurred to me that the cause of all this distress could be no other than than extravasated blood from some vessel or vessels which had given way, and that, by making a free opening, I should stand some chance of diminishing my patient's sufferings, and, possibly, of putting a stop to the mischief, which was every hour growing more extensive and threatening. Impressed with this idea, I proposed the operation, to which she readily assented, and, soon after five in the morning, I made a longitudinal incision with a lancet, nearly five inches in extent, in the most prominent part of the tumour. On passing a finger between the edges of the incision, a good deal of coagulated blood was perceived, which was broken down and extracted; immediately after which there rushed out a pretty large quantity in a fluid state; this stream, however, was not considerable enough to occasion faintness, and, indeed, very soon became trifling. As soon as the opening was made, the patient expressed a sense of relief, and, in about fifteen minutes, fell into a dose; she continued, with some intermissions, in a dosing state till about half past nine, when the tumour was much diminished, particularly at its upper part. At 2 P. M. the tumour was still less, the patient tolerably easy, and able to bear her legs closed.

The discolouration of the loose cellular membrane had now exactly the appearance of ecchymosis from contusion.

The use of the common fomentation was directed every six hours, the unguentum ceræ, spread on lint, and a bread and milk poultice. Matters went on so well, that on the 3d of September the tumour and pain were inconsiderable; but as there was evidently extravasated blood still within the tumour, and the air of the chamber was grown very offensive, a poultice, made with small beer and fine oatmeal, with the addition of a large spoon full of yeast, was applied, and renewed every three hours. Coagula came away in every poultice, but after four days no more were visible; the edges of the wound had likewise thrown off all their sloughs, and looked clean and healthy; and the air of the chamber had lost its offensiveness. From this time the unguentum ceræ only was made use of as dressing.

A clyster was, at first, administered every day; afterwards every other day. Although the urine had frequently drizzled away, and apparently in large quantities, yet the distension of the bladder became so great on the 1st of September, that it appeared necessary to introduce the catheter, when nearly five pints were drawn off; on the following day a full quart was again drawn off; but, on the third, third, she was able to raise herself on her knees, and to pass a fair quantity.

The woman complained of great pain in both legs from the commencement of the tumour, and it gradually went off as the tumour subsided.

The milk-fever was moderate, and every thing relative to the lochial discharge, as well as the breasts, perfectly natural, and in no respect different from what it generally is.

On the sixth day she bore being removed to a couch whilst her bed was made; and, on the twenty-first, the wound was completely healed, and the labinm reduced to its natural size and appearance.

